# Exenatide improves hepatocyte insulin resistance induced by different regional adipose tissue

**DOI:** 10.3389/fendo.2022.1012904

**Published:** 2022-09-29

**Authors:** Chuanmin Bai, Yujun Wang, Zhi Niu, Yaxin Guan, Jingshan Huang, Xin Nian, Fan Zuo, Juan Zhao, Tsutomu Kazumi, Bin Wu

**Affiliations:** ^1^ Department of Endocrinology, First Affiliated Hospital, Kunming Medical University, Kunming, China; ^2^ School of Computing, University of South Alabama, Mobile, AL, United States; ^3^ Open Research Center for Studying of Lifestyle−Related Diseases, Mukogawa Women’s University, Nishinomiya, Japan; ^4^ Research Institute for Nutrition Sciences, Mukogawa Women’s University, Nishinomiya, Japan; ^5^ Department of Medicine, Kohnan Kakogawa Hospital, Kakogawa, Japan

**Keywords:** obesity, adipose tissue, GLP-1, incretin, exenatide, insulin resistance

## Abstract

Obesity is resulted from energy surplus and is characterized by abnormal adipose tissue accumulation and/or distribution. Adipokines secreted by different regional adipose tissue can induce changes in key proteins of the insulin signaling pathway in hepatocytes and result in impaired hepatic glucose metabolism. This study aimed to investigate whether exenatide affects key proteins of IRS2/PI3K/Akt2 signaling pathway in hepatocytes altered by the different regional fat depots. Six non-obese patients without endocrine diseases were selected as the research subjects. Their subcutaneous adipose tissue (SAT) and visceral adipose tissue (VAT)were co-cultured with HepG2 cells in the transwell chamber. In the presence or absence of exenatide, adipokines content in the supernatant of each experimental group was detected by ELISA. In addition, HepG2 cells in each co-culture group with and without insulin were collected, and the expression of key proteins IRS2, p-IRS2(S731), PI3K-p85, Akt2, and p-Akt2(S473) was detected by western blotting (WB). The results showed that the adipokines IL-8, MCP-1, VEGF, and sTNFR2 in the supernatant of HepG2 cells induced by different regional adipose tissue were significantly higher than those in the HepG2 group, and VAT released more adipokines than SAT. Furthermore, these adipokines were significantly inhibited by exenatide. Importantly, the different regional fat depot affects the IRS2/PI3K/Akt2 insulin signaling pathway of hepatocytes. Exenatide can up-regulate the expression of hepatocyte proteins IRS2, PI3K-p85, p-Akt2(S731) inhibited by adipose tissue, and down-regulate the expression of hepatocyte proteins p-IRS2(S731) promoted by adipose tissue. The effect of VAT on the expression of these key proteins in hepatocytes is more significant than that of SAT. But there was no statistical difference in the expression of Akt2 protein among each experimental group, suggesting that exenatide has no influence on the expression of Akt2 protein in hepatocytes. In conclusion, exenatide may improve hepatic insulin resistance (IR) by inhibiting adipokines and regulating the expression of key proteins in the IRS2/PI3K/Akt2 pathway.

## 1 Introduction

The global prevalence of obesity has increased over recent decades and it is now considered a major health problem (1, 2). Obesity mainly manifests in the excessive accumulation and abnormal distribution of adipose tissue in the body. Adipose tissue present around abdominal viscera in mesentery and momentum, known as visceral adipose tissues (VAT) is more insulin-resistant than adipose tissue present in subcutaneous areas (subcutaneous adipose tissue, SAT) (1). VAT is more likely to cause obesity-related metabolic disorders than SAT (3). Compared with SAT, VAT contains more macrophages and has a greater ability to generate free fatty acids (FFA) and uptake glucose (4, 5). Moreover, because of its anatomical position, VAT venous blood is drained directly to the liver through the portal vein, this contrasts with SAT where venous drainage is through systemic veins. The portal drainage of VAT causes the direct influx of adipokines and FFA into the liver, which may affect the action of hepatic insulin and lead to IR (6–8).

Insulin plays an important role in the regulation of plasma glucose homeostasis (9). The target tissues of insulin action mainly include the liver, skeletal muscle, and adipose tissue. About 60% of insulin enters the liver from the portal system and then passes through the “ first-pass effect “ of the liver to surrounding tissues (10, 11). Therefore, the liver is exposed to the highest concentration of insulin which makes it as the central organ of insulin action and glucose metabolism.

Obese patients are prone to hyperglycemia, which may be related to adipokines interfering with hepatic insulin signaling pathways (12). Insulin secreted into the blood binds to the insulin receptor on hepatocytes, resulting in a change in the receptor conformation, and autophosphorylation activates tyrosine protein kinase, which activates insulin receptor substrate (IRS) containing tyrosine residues through the catalytic domain of the intracellular segment. Abnormalities in the number, affinity and structure of IRS may lead to the occurrence of IR. Protein IRS mainly includes IRS1, IRS2, IRS3, and IRS4. Multiple studies on IRS2-/- mice have confirmed that IRS2 plays an important role in regulating glucose homeostasis. Activated IRS2 can further activate phosphatidylinositol3-kinase (PI3K), which phosphorylates phosphatidylinositol-4,5-bisphosphate (PIP2), resulting in the formation of phosphatidylinositol-3,4,5-trisphosphate (PIP3) at the plasma membrane. PIP3 then recruits protein kinase B (Akt) and pyruvate dehydrogenase kinase 1 (PDK1) from the cytoplasm to the plasma membrane. PDK1 phosphorylates the Thr308 site of Akt, followed by pyruvate dehydrogenase kinase 2 (PDK2) phosphorylation of the Ser473 site of Akt, and the overall activation of AKt further cascades the activation of downstream signaling pathways. In fact, the hepatocyte insulin signaling pathway is an extremely complex cascade activation system (13–16). Although Akt contains three protein isoforms of Akt1, Akt2 and Akt3. Metabolic activities such as glycogen synthesis and glucose uptake are closely related to Akt2. IRS2/PI3K/Akt2 signaling pathway plays an important role in glucose metabolism.

In our previous study, adipose tissue of different regions and human hepatocytes were co-cultured in transwell chambers. The results showed that both VAT and SAT affected the key proteins of the insulin signaling pathway and glucose metabolism in hepatocytes, and the effect of VAT was more pronounced than that of SAT(unpublished data). This may be due to the difference in adipokines secreted by VAT and SAT, resulting in different expressions of key proteins in the hepatic insulin signaling pathway, which in turn affects hepatic uptake of glucose and synthesis of glycogen.

Glucagon-like peptide-1 (GLP-1) is an incretin hormone secreted into the blood by the intestines under the stimulation of food, especially carbohydrates. It acts by specifically activating GLP-1 receptors and inhibits the rise of postprandial blood sugar (17–19). Exenatide (EXE), the first clinically used GLP-1 receptor agonist (GLP-1RA), is a hypoglycemic agent with novel mechanisms developed in the field of type 2 diabetes mellitus (T2DM) treatment in recent years. It has the functions of improving IR, protecting pancreatic Β cells, lowering blood pressure, lowering lipids, lowering body mass index (BMI) and fat accumulation (20).

Numerous studies have shown that the glucagon-like peptide-1 receptor (GLP-1R) is mainly expressed in the pancreas, brain, adipose tissue, muscle, heart, kidney, lung, stomach, and liver (21). Junling Liu et al. further confirmed that GLP-1R protein and mRNA were expressed in HepG2 cells (22). Moreover, Rajaa El Bekay et al. obtained evidence for the presence of GLP-1R in adipose tissue. GLP-1R mRNA and protein levels are increased in VAT from morbidly obese patients with a high degree of IR (23).

Hepatic IR plays a pivotal role in the pathogenesis of diabetes. Previous studies have not reported whether exenatide influences hepatic IR induced by different regional adipose tissue. Thus, this study investigated the effect of exenatide on the expression of key proteins in the hepatic IRS2/PI3K/Akt2 signaling pathway induced by different regional fat depots of the human body, with purpose to explore whether adipose tissue -related IR might be alleviated by exenatide and provide experimental evidences for further understanding the mechanisms of GLP-1RA.

## 2 Materials and methods

### 2.1 Study participants

All the subjects were Han population who have lived in Yunnan province, China for a long time (>10 years) and were not related to each other. The average age was 71 (24, 84) years old, and the average BMI was 21.35 (17.80, 24.03) kg/m^2^. We collected about 3g of VAT and SAT of 6 patients who underwent laparotomy in the Department of Gastrointestinal Surgery of the First Affiliated Hospital of Kunming Medical University. Three subjects were randomly allocated to the LDH activity examination and adipokines assay, and the remaining three subjects were allocated to the expression of key proteins of IRS2/PI3K/Akt insulin signaling pathway. Patients were excluded if they had cardiovascular disease, chronic renal diseases, endocrine disease (such as diabetes mellitus, non-alcoholic fatty liver disease, hypothyroidism, and Cushing syndrome), infectious disease or had undergone recent weight change (>2kg in 3 months). All participants gave their written informed consent. The study was reviewed and approved by the Ethics and Research Committee of the First affiliated hospital of Kunming Medical University.

HepG2 of the human liver tumor cell line was purchased from the Cell Bank of Kunming Institute of Animal Science.

### 2.2 Adipose tissue culture

On a sterile ultra-clean workbench, we carefully removed the blood vessels and fibrous connective tissue in the sample adipose tissue, and then cut the adipose tissue into small pieces so that each adipose tissue is about 100mg. Rinse with PBS solution and inoculate into 12-well culture plates. After adding 1ml of conditioned medium, the adipose tissue was cultured in a 37°C, 5% CO2 cell culture incubator. The medium was changed every 24 hours, and it was used after 4 days of culture.

### 2.3 Establishment of co-culture system of HepG2 cells and adipose tissue

The VAT or SAT was co-cultured with HepG2 cells using a transwell chamber (membrane pore size 0.4μm). Firstly, HepG2 cells were evenly seeded into 6-well culture plates, resulting in 5×10^4^ cells per well, with 3 replicates per group. DMEM(H) complete medium was added to each well. Cells were cultured at 37°C with 5% CO_2_. Secondly, when the cell confluence reaches about 50%, the old medium is aspirated and replaced with DMEM (H) basal culture. Thirdly, transwell chambers were placed on the HepG2 cells. The VAT or SAT that can stably release adipokines on the 4th day of culture were washed twice with DMEM (H) basal culture and then placed on transwell chambers respectively. The plates were placed in a 37°C and 5% CO_2_ incubator for 48 hours, which constituted the HepG2 + VAT group and the HepG2 + SAT group. Since the pore size of the transwell chamber is only 0.4μm, the adipose tissue (AT) in the upper chamber and the HepG2 cells in the lower chamber don’t contact each other, but the secretions of the adipose tissue can enter the lower chamber through the permeability of the transwell chamber. Subsequent experiments were grouped according to the purpose of the experiment.

### 2.4 Detection of LDH in the supernatant of the co-culture system

The groups of lactate dehydrogenase (LDH) activity assays are as follows: ①HepG2 group, ②HepG2+VAT group, ③HepG2+SAT group, ④HepG2+VAT+EXE group, ⑤HepG2+SAT+EXE group, ⑥ HepG2+TritonX-100 group. After 48 hours of co-culture in groups ① to ⑤, the transwell chamber and adipose tissue were removed, and the HepG2 cells were centrifuged to collect the supernatant of each group. HepG2 cells of ⑥groups were cultured for 43 hours, treated with 0.4% TritonX-100 for 5 hours, and the HepG2 cells were centrifuged to collect the supernatant. The LDH concentration of each experimental group was detected according to the instructions of the Human Lactate Dehydrogenase (LDH) ELISA Kit (Enzyme-linked Biotechnology Co., Ltd, Shanghai, China). The experimental results showed that the HepG2 cells in the above groups ① to ⑤could still maintain a certain activity after 48 hours. Therefore, follow-up experiments can be performed.

### 2.5 Detection of adipokines in the supernatant of the co-culture system by ELISA

The grouping is the same as the above-mentioned in section 2.3. Our previous study on the differences of adipokines secreted by of adipose tissue at different sites has confirmed that interleukin-8 (IL-8), monocyte chemoattractant protein-1 (MCP-1), vascular endothelial growth factor (VEGF), and soluble tumor necrosis factor receptor II (sTNFR2) are highly expressed in VAT and low in SAT. To further verify whether exenatide influences the expression of adipokines under co-culture conditions, this part of the experiment was designed. After 48 hours of co-cultivation, adipose tissue and the transwell chamber were removed, and the HepG2 cells were centrifuged to collect the supernatant of each experimental group. The operation was performed according to the instructions of the respective detection kits for Human IL-8 ELISA Kit (Enzyme-linked Biotechnology Co., Ltd, Shanghai, China), Human Monocyte chemotactic protein 1(MCP-1) ELISA Kit (Enzyme-linked Biotechnology Co., Ltd, Shanghai, China), Human Vascular Endothelial cell Growth Factor (VEGF) ELISA Kit (Enzyme-linked Biotechnology Co., Ltd, Shanghai, China), and Human soluble Tumor Necrosis Factor receptor 2 (sTNFR2) ELISA Kit (Enzyme-linked Biotechnology Co., Ltd, Shanghai, China).

A standard curve was made according to the standard density and OD value in ELISA calc software, according to the obtained regression equation, the content of adipokines in the sample was calculated.

### 2.6 Detection of key proteins of insulin signaling pathway IRS2/PI3K/AKt by western blot

The key protein assays for the IRS2/PI3K/Akt2 signaling pathway were grouped as follows: ①HepG2 group, ②HepG2+VAT group, ③HepG2+SAT group, ④HepG2+VAT+EXE group, ⑤HepG2+SAT+EXE group, ⑥HepG2+insulin group, ⑦HepG2+VAT+insulin group, ⑧HepG2+SAT+insulin group, ⑨HepG2+VAT+EXE+insulin group, ⑩HepG2+SAT+EXE+insulin group. In groups ① to ⑤, HepG2 cells and adipose tissue were co-cultured for 48 hours. After removing the VAT or SAT and the transwell chamber, the medium was replaced with a new DMEM high-glucose basal medium for 10 minutes. In groups ⑥ to ⑩, the transwell chamber and adipose tissue were removed after co-culturing HepG2 cells with VAT or SAT for 48 hours. The medium was replaced with DMEM high-glucose basal medium containing 10 nmol/L insulin for 10 minutes. HepG2 cells from each group were collected. The total protein of HepG2 cells in each group was extracted and the protein concentration was determined. WB detected the protein expression of IRS2, p-IRS2(S731), PI3K-p85, Akt2, p-Akt2(S473).

First, protein samples were to be prepared. Aspirated the old medium, washed the cells twice with PBS, added 300μl of RIPA lysis solution containing 30μl of protease inhibitors to each well (6-well plate), and let stand on ice for 10 min. The cells were pipetted down with a liquid dispenser and collected into a 1.5ml centrifuge tube. Next, the total protein concentration of the cells was determined. Centrifuge at 12000rpm/min for 10min at 4°C, collect the supernatant into a new 1.5ml centrifuge tube, store on ice, and measure the total protein concentration of cells in each group by BCA Protein Assay Kit (Beyotime Biotechnology Co., Ltd, Shanghai, China). Finally, SDS-PAGE electrophoresis was performed. The main reagents in the experiment were marked in **Table 1**.

**Table 1:** Reagent information required for western blot.

Reagent	Manufacture	Dilution	protein(kD)
anti-IRS2 antibody [EPR904(2)]	abcam	1:1000	160
Anti-IRS2 (phospho S731) antibody	abcam	1:5000	160
PI3 Kinase p85 (19H8) Rabbit mAb	CST	1:1000	85
Akt2 (5B5) Rabbit mAb	CST	1:1000	60
Phospho-Akt (Ser473) (D9E) XP^®^ Rabbit mAb	CST	1:1000	60
GAPDH Rabbit pAb	abmart	1:1000	37
Anti-rabbit IgG, HRP-linked Antibody	CST	1:2000	—

The primary and secondary antibodies required for western blot were diluted with TBST to appropriate concentrations before use.

### 2.7 Statistical analyses

Quantification of WB bands was performed with ImageJ. SPSS 17.0 statistical software was used to analyze the data, and GraphPad Prism 8 was used for graphing. The experimental data were tested for normality by Shapiro-Wilk and homogeneity of variances by Levene. In the measurement data of multi-group random block design, the random block design analysis of variance is used for the normal distribution. If the variances in each group were equal, LSD-t was used for pairwise comparison. If the variance of each group is not uniform, the rank sum test of the data of random block design is used. For the measurement data of two independent samples that obey the normal distribution, the independent sample t test is used for homogeneous variances, and the approximate t’ test is used for unequal variances. For the measurement data of a multi-group randomized block design that does not obey the normal distribution, the Kruskal-Willis rank-sum test was used for data comparison. If the measurement data of the two independent samples did not obey the normal distribution, the data were compared using the Wilcoxon rank sum test. Normally distributed data were statistically described as mean ± to standard error (mean±SEM). Non-normally distributed data were statistically described as medians (interquartile range). Statistical significance was set at P< 0.05.

## 3 Results

### 3.1 Determination of LDH level

**Figure 1:**
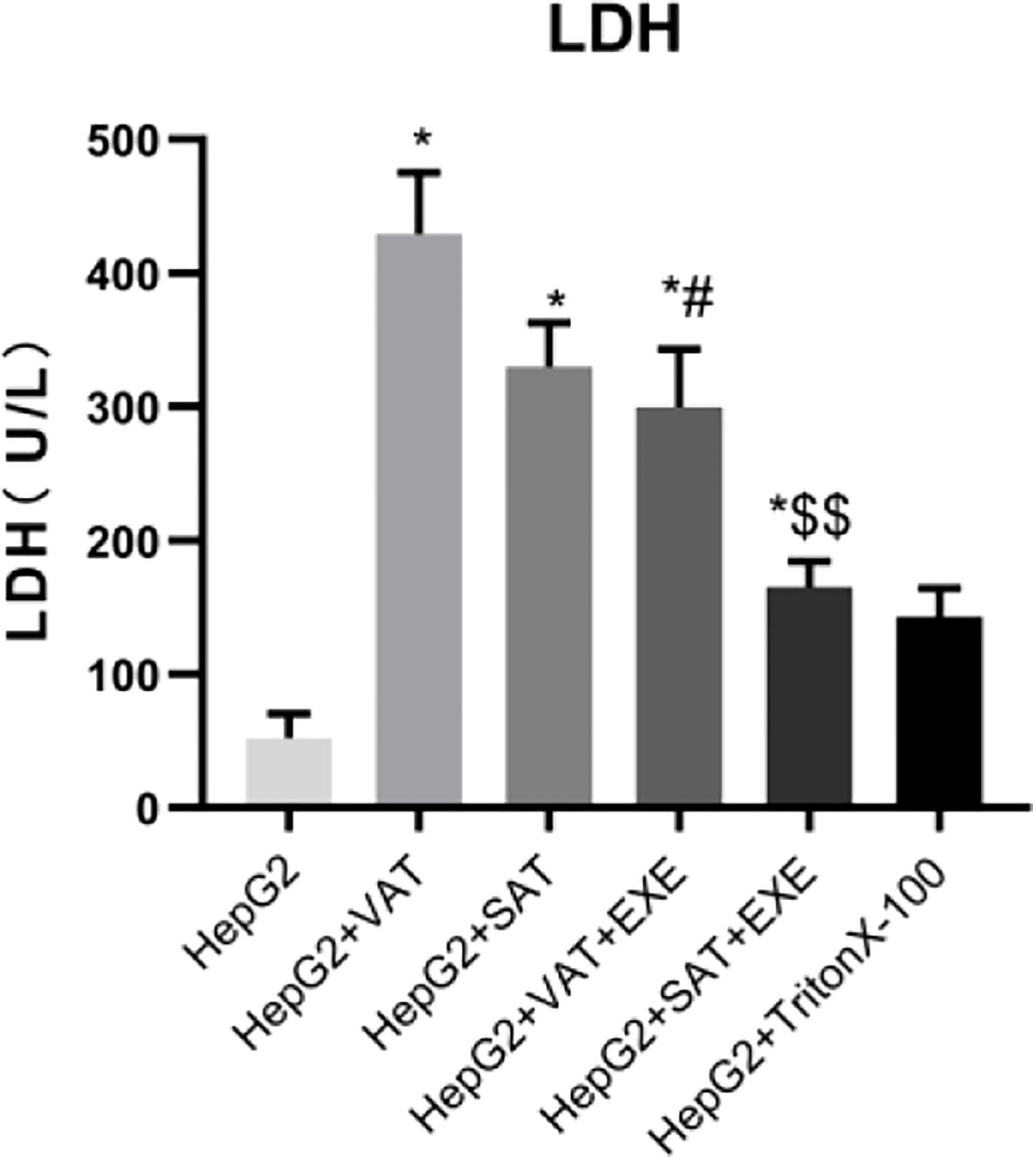
The content of LDH in supernatant of each group. Data are means ± SEM, n = 3/group, *P < 0.05 vs. HepG2 group; ^#^P < 0.05 vs. HepG2 + VAT group; ^$$^P < 0.01 vs. HepG2+SAT group.

**Figure 2:**
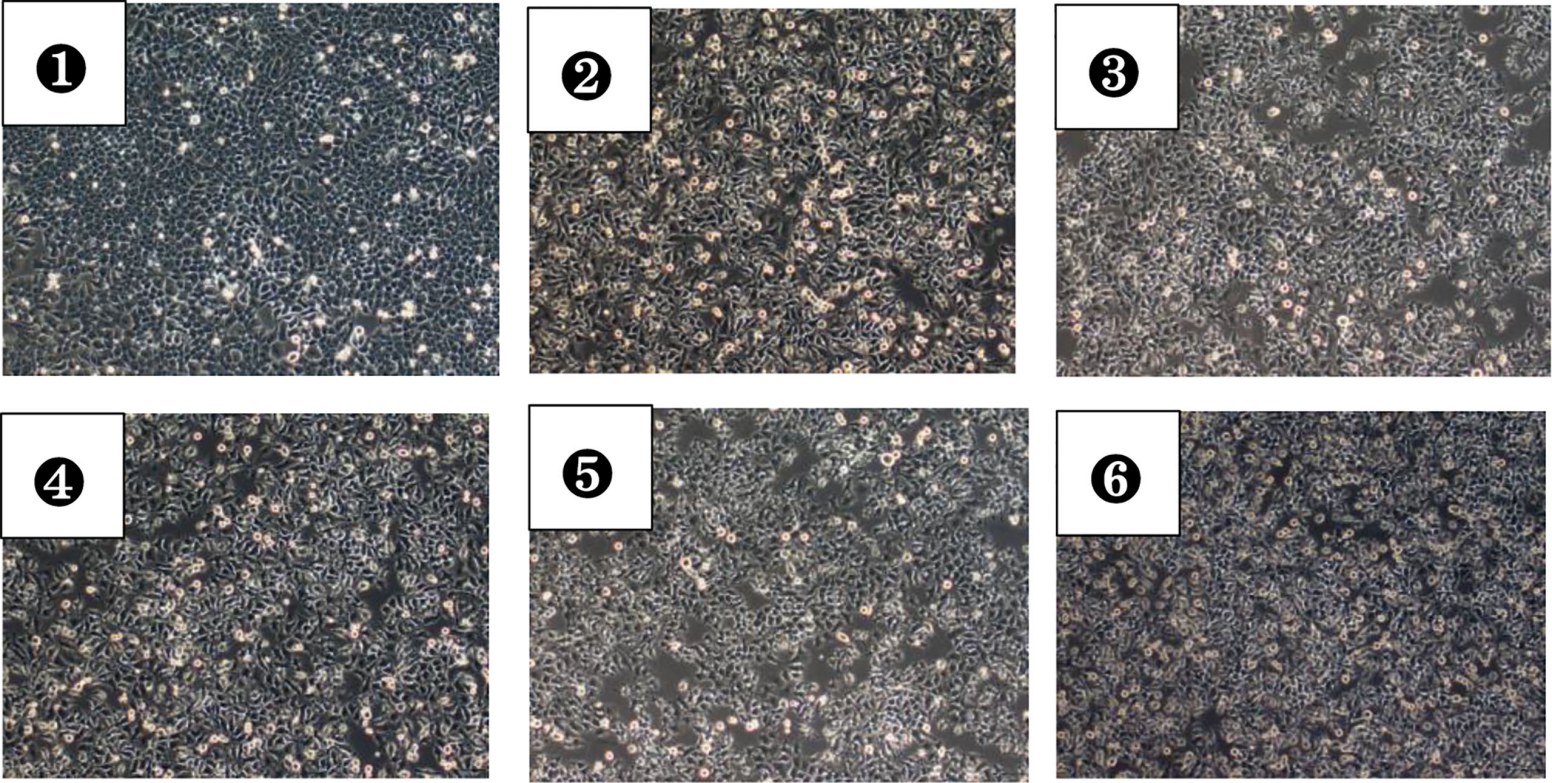
HepG2 cells in each group after 48 hours of culture (magnification, ×100). ①A photo of cell growth in the HepG2 group; ②A photo of cell growth in the HepG2+VAT group; ③A photo of cell growth in the HepG2+SAT group; ④A photo of cell growth in the HepG2+VAT+EXE group; ⑤A photo of cell growth in the HepG2+SAT+EXE group; ⑥A photo of cell growth in the HepG2+TritonX-100 group.

Compared with HepG2 group, LDH levels in the supernatant of HepG2+VAT group, HepG2+SAT group, HepG2+VAT+EXE group, HepG2+SAT+EXE group, and HepG2+TritonX-100 group were all increased (p<0.05). The LDH level of HepG2+VAT+EXE group was lower than that of HepG2+VAT group (299.67± 43.54 U/L vs. 429.34 ± 45.51U/L, p<0.05). Similarly, compared with the HepG2+SAT group, the LDH level of the HepG2+SAT+EXE group was decreased (329.70 ± 32.95U/L vs. 164.92 ± 19.53U/L, p<0.05). According to the LDH content (**Figure 1**) and growth photos of HepG2 cells (**Figure 2**) in each group, the HepG2 cells still have certain viability, and subsequent experiments can be carried out.

### 3.2 Exenatide affects adipokines secreted by adipose tissue at different region

Our previous studies have confirmed that human VAT releases IL-8, MCP-1, VEGF and sTNFR2 more than SAT. However, we still do not know whether there are changes in the release of cytokines from adipose tissue in the co-culture system of hepatocytes, and what effect exenatide has on these adipokines. Therefore, we further explored the content of adipokines in the supernatant of each group under the co-culture system.

#### 3.2.1 Exenatide inhibits IL-8 secretion under co-culture conditions

**Figure 3:**
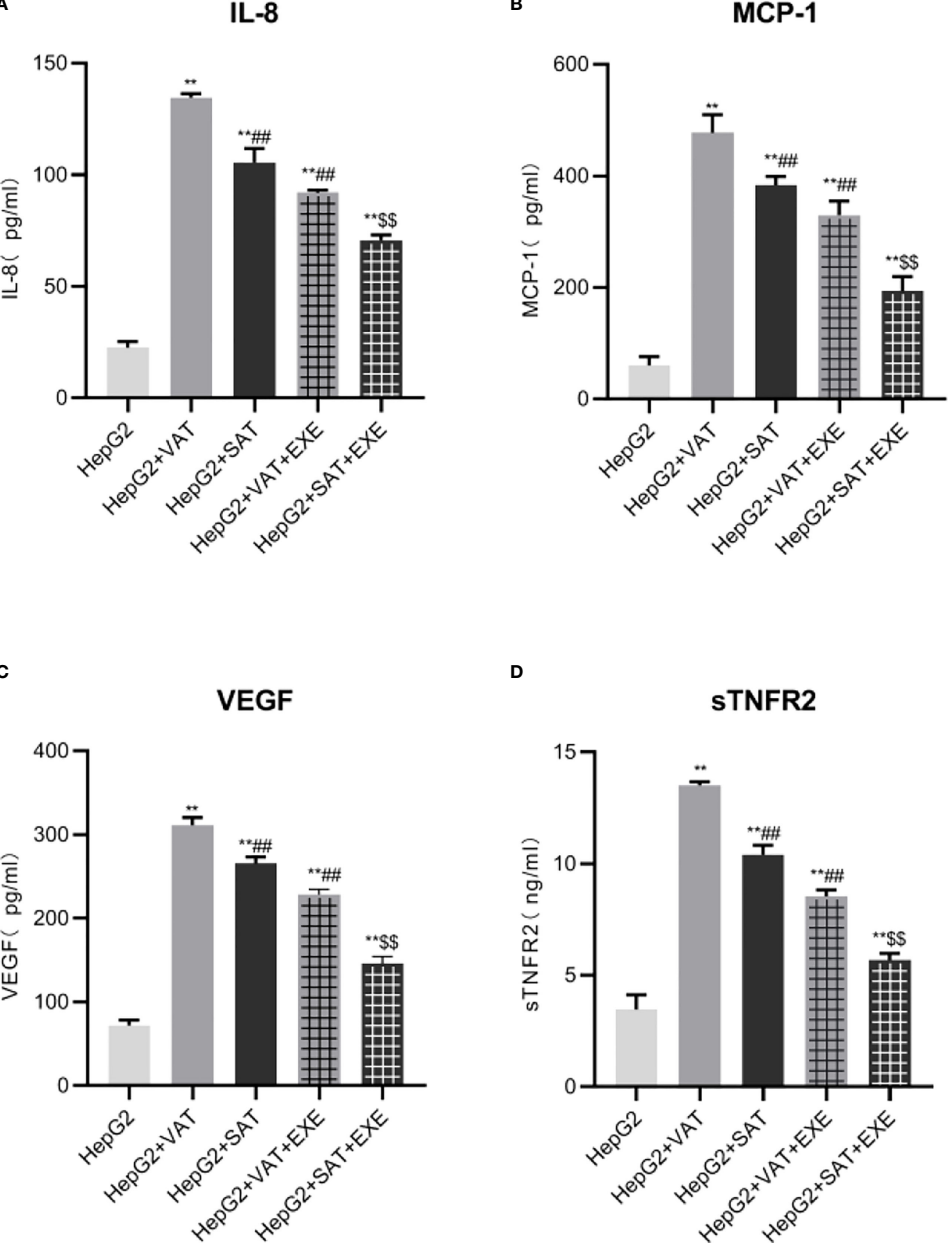
Exenatide inhibits adipokines secreted by adipose tissue at different sites. **(A)**The content of IL-8 in the supernatant of each group. **(B)** The content of MCP-1 in the supernatant of each group. **(C)** The content of VEGF in the supernatant of each group. **(D)** The content of sTNFR2 in the supernatant of each group. Data are means ± SEM, n = 3/group, **P < 0.01 vs. HepG2 group; ^##^P < 0.01 vs. HepG2+VAT group; ^$$^P < 0.01 vs. HepG2+SAT group.

Compared with the HepG2 group, the levels of IL-8 in the HepG2+VAT group, HepG2+SAT group, HepG2+VAT+EXE group and HepG2+SAT+EXE group were all elevated (p<0.01). Notably, the levels of IL-8 in the HepG2+VAT group was higher than that in the HepG2+SAT group (134.60 ± 1.72pg/mL vs. 105.42 ± 6.36pg/mL, p<0.01). Compared with the HepG2+VAT group, the IL-8 in the HepG2+VAT+EXE group was decreased (134.60 ± 1.72pg/mL vs. 91.99 ± 1.15pg/mL, p<0.01). IL-8 was also decreased in the HepG2+SAT+EXE group compared with the HepG2+SAT group (105.42 ± 6.36pg/m vs. 70.63 ± 2.36pg/m, p<0.01). As expected, VAT secreted IL-8 more than SAT under co-culture conditions (**Figure 3A**). IL-8 secreted by adipose tissue in different parts can be inhibited by exenatide.

#### 3.2.2 Exenatide inhibits MCP-1 secretion under co-culture conditions

HepG2+VAT group, HepG2+SAT group, HepG2+VAT+EXE group and HepG2+SAT+EXE group secret more MCP-1 than HepG2 group(p<0.01). The level of MCP-1 in the HepG2+VAT group was higher than that in the HepG2+SAT group (477.38 ± 32.23pg/mL vs. 383.43 ± 15.28pg/mL, p<0.01). Exenatide inhibits MCP-1 secretion under co-culture conditions. Specifically, the level of MCP-1 in HepG2+VAT+EXE group was lower than that in HepG2+VAT group (329.52 ± 25.07pg/mL vs. 477.38 ± 32.23pg/mL, p<0.01). Similarly, MCP-1 levels were also decreased in the HepG2+SAT+EXE group compared to the HepG2+SAT group (193.44 ± 25.89pg/m vs. 383.43 ± 15.28pg/m, p<0.01) (**Figure 3B**).

#### 3.2.3 Exenatide inhibits VEGF secretion under co-culture conditions

Compared with the HepG2 group, the levels of VEGF in the HepG2+VAT group, HepG2+SAT group, HepG2+VAT+EXE group and HepG2+SAT+EXE group were all elevated (p<0.01). VAT secretes more VEGF than SAT under co-culture conditions (311.38 ± 9.17pg/mL vs. 265.75 ± 7.74pg/mL, p<0.01). Exenatide inhibits VEGF secreted by different regional adipose tissue. Compared with the HepG2+VAT group, the VEGF in the HepG2+VAT+EXE group was decreased (311.38 ± 9.17pg/mL vs. 228.31 ± 6.34pg/mL, p<0.01). Compared with the HepG2+SAT group, the VEGF in the HepG2+SAT+EXE group was decreased (265.75 ± 7.74pg/mL vs. 145.76 ± 8.59pg/mL, p<0.01) (**Figure 3C**).

#### 3.2.4 Exenatide inhibits sTNFR2 secretion under co-culture conditions

Different parts of adipose tissue secreted sTNFR2 differently. HepG2+VAT group, HepG2+SAT group, HepG2+VAT+EXE group and HepG2+SAT+EXE group secret more sTNFR2 than HepG2 group(p<0.01). The level of sTNFR2 in the HepG2+SAT group was lower than that in the HepG2+VAT group (10.40 ± 0.43ng/mL vs. 13.50 ± 0.18ng/mL, p<0.01). Exenatide inhibits sTNFR2 secretion under co-culture conditions. Compared with the HepG2+VAT group, the sTNFR2 in the HepG2+VAT+EXE group was decreased (13.50 ± 0.18ng/mL vs. 8.53 ± 0.30ng/mL, p<0.01). sTNFR2 was also decreased in the HepG2+SAT+EXE group compared with the HepG2+SAT group (10.40 ± 0.43ng/mL vs. 5.69 ± 0.30ng/mL, p<0.01) (**Figure 3D**).

### 3.3 Effects of exenatide on key proteins of IRS2/PI3K/Akt2 signaling pathway in hepatocytes induced by different regional adipose tissue

**Figure 4:**
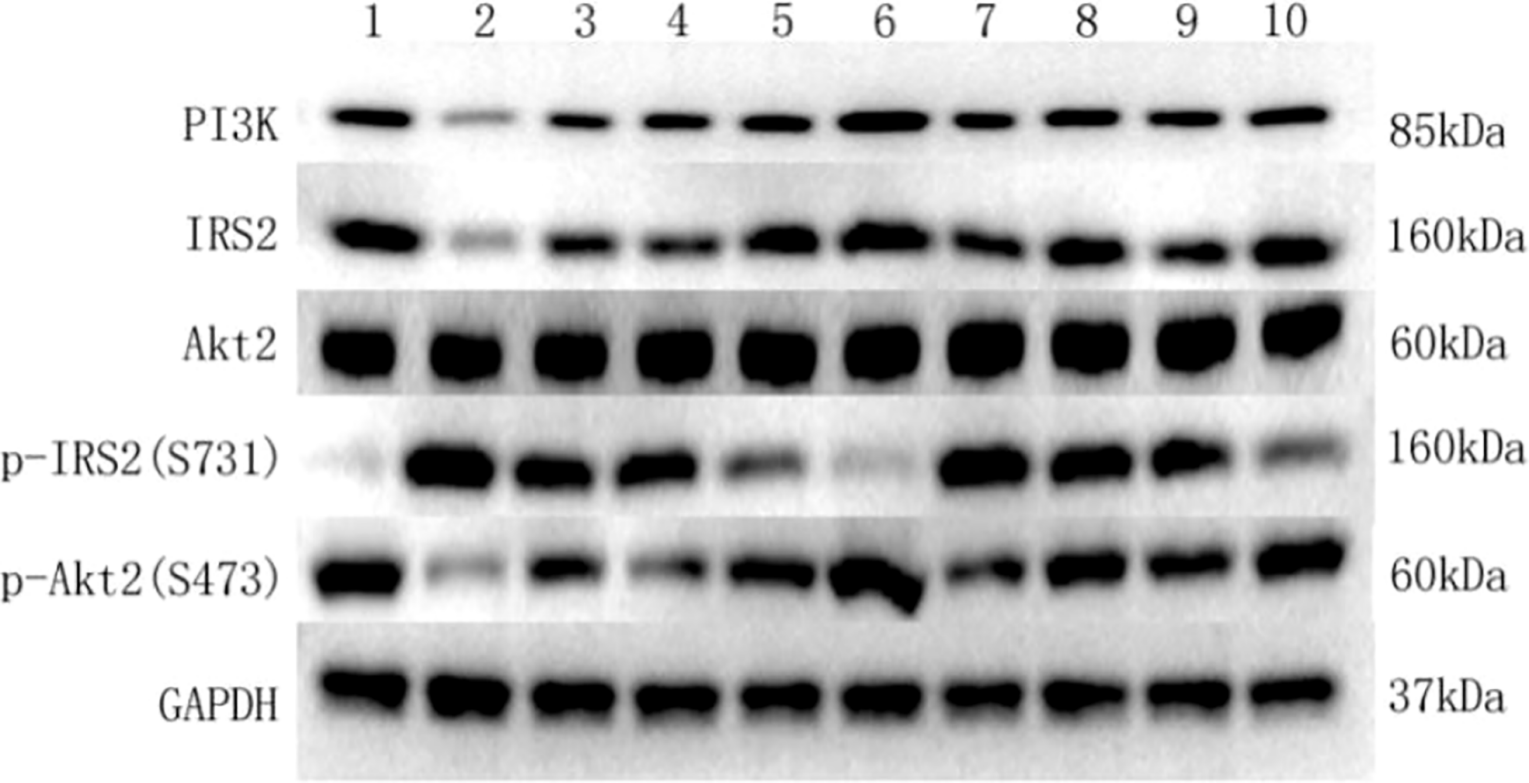
Key protein bands of insulin signaling pathway detected by western blot in each group. (1) Key protein bands in the HepG2 group. (2) Key protein bands in the HepG2+VAT group. (3) Key protein bands in the HepG2+SAT group. (4) Key protein bands in the HepG2+VAT+EXE group. (5) Key protein bands in the HepG2+SAT+EXE group. (6) Key protein bands in the HepG2+insulin group. (7) Key protein bands in the HepG2+VAT +insulin group. (8) Key protein bands in the HepG2+SAT +insulin group. (9) Key protein bands in the HepG2+VAT+EXE +insulin group. (10) Key protein bands in the HepG2+SAT+EXE +insulin group.

Western blot was used to detect the protein expressions of IRS2, p-IRS2(S731), PI3K-p85, Akt2, and p-AKt2(S473) in HepG2 cells of each culture group. A clear IRS2 band can be seen at the protein molecular weight of 160KD. A clear p-IRS2(S731) band can be seen at the protein molecular weight of 160KD. A clear PI3K-p85 band can be seen at the protein molecular weight of 85KD. A clear Akt2 band can be seen at the protein molecular weight of 60KD. A clear p-Akt2(S473) band can be seen at the protein molecular weight of 60KD. A GAPDH band can be seen at the protein molecular weight of 37KD (**Figure 4**).

#### 3.3.1 Exenatide promotes the expression of IRS2 protein in HepG2 cells

**Figure 5:**
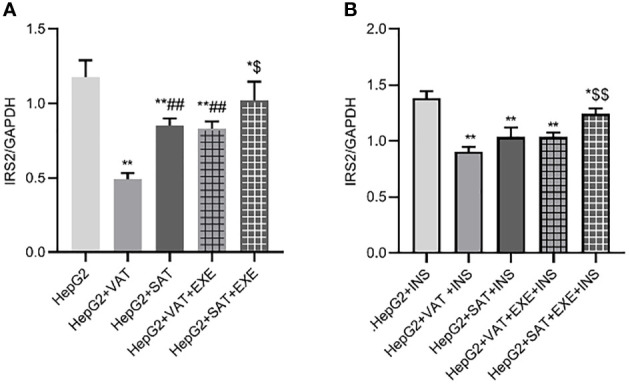
The influence of exenatide on the expression of IRS2 protein in hepatocytes conducted by different regional adipose tissue. **(A)** Expression of IRS2 protein in HepG2 cells of each group. Data are means ± SEM, n = 3/group, *P < 0.05, **P < 0.01 vs. HepG2 group; ^##^P < 0.01 vs. HepG2+VAT group; ^$^P< 0.05 vs. HepG2+SAT group. **(B)** Expression of IRS2 protein in HepG2 cells of each group under the action of insulin. Data are means ± SEM, n = 3/group, *P < 0.05, **P < 0.01 vs. HepG2+INS group; ^$$^P < 0.01 vs. HepG2+SAT+INS group.

Adipose tissue can inhibit the expression of IRS2 protein in HepG2 cells. Specifically, compared with HepG2 group, the expression of IRS2 in HepG2+VAT group (1.18 ± 0.11/GAPDH vs. 0.49 ± 0.04/GAPDH, p<0.01) and HepG2+SAT group (1.18 ± 0.11/GAPDH vs. 0.85 ± 0.05/GAPDH, p<0.01) was decreased. HepG2+VAT group inhibited IRS2 protein expression more than HepG2+SAT group (0.49 ± 0.04/GAPDH vs. 0.85 ± 0.05/GAPDH, p<0.01). Furthermore, exenatide improves the inhibitory effect of adipose tissue on IRS2 protein in HepG2 cells. Compared with the HepG2+VAT group, the expression of IRS2 protein was increased in the HepG2+VAT+EXE group (0.49 ± 0.04/GAPDH vs. 0.83 ± 0.05/GAPDH, p<0.01). The protein of IRS2 was also increased in the HepG2+SAT+EXE group compared with the HepG2+SAT group (1.02 ± 0.13/GAPDH vs. 0.85 ± 0.05/GAPDH, p<0.05) (**Figure 5A**).

To further explore the effect of insulin on IRS2 protein, we added insulin to each co-culture group. Compared with HepG2+insulin group, the expression of IRS2 protein in HepG2+VAT+insulin group, HepG2+SAT+insulin, HepG2+VAT+EXE+insulin group and HepG2+SAT+EXE+insulin group was decreased(p<0.05). Compared with HepG2+VAT+insulin group, the expression of IRS2 in HepG2+SAT+insulin group was higher, but the difference was not statistically significant (0.90 ± 0.05/GAPDH vs. 1.04 ± 0.08/GAPDH, p>0.05). The expression of IRS2 protein in HepG2+VAT+insulin group was lower than that in HepG2+VAT+EXE+insulin group, but there was also no statistical difference (0.90 ± 0.05/GAPDH vs. 1.03 ± 0.04/GAPDH, p>0.05). Compared with HepG2+SAT+insulin group, the expression of IRS2 protein in HepG2+SAT+EXE+insulin group was significantly increased (1.04 ± 0.08/GAPDH vs. 1.24 ± 0.05/GAPDH, p<0.01) (**Figure 5B**).

#### 3.3.2 Exenatide inhibits the expression of p-IRS2(S731) protein in HepG2 cells

**Figure 6:**
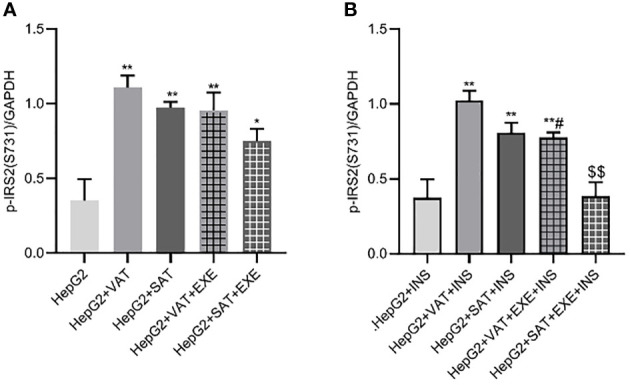
The influence of exenatide on the expression of p-IRS2(S731) protein in hepatocytes conducted by different regional adipose tissue. **(A)** Expression of p-IRS2(S731) protein in HepG2 cells of each group. Data are means ± SEM, n = 3/group, *P < 0.05, **P < 0.01 vs. HepG2 group. **(B)** Expression of p-IRS2(S731) protein in HepG2 cells of each group under the action of insulin. Data are means ± SEM, n = 3/group, **P < 0.01 vs. HepG2+INS group; ^#^P < 0.05 vs. HepG2+VAT+INS group; ^$$^P < 0.01 vs. HepG2+SAT+INS group.

Adipose tissue can promote the expression of p-IRS2(S731) protein in HepG2 cells. Compared with HepG2 group, the expression of p-IRS2(S731) protein in HepG2+VAT group (0.35 ± 0.15/GAPDH vs. 1.11 ± 0.08/GAPDH, p<0.01) and HepG2+SAT group (0.35 ± 0.15/GAPDH vs. 0.97 ± 0.04/GAPDH, p<0.01) was elevated. Compared with the HepG2+VAT group, the expression of p-IRS2(S731) protein in the HepG2+SAT group was lower (1.11 ± 0.08/GAPDH vs. 0.97 ± 0.04/GAPDH, p>0.05), but the difference was not statistically significant. The expression of p-IRS2(S731) protein in the HepG2+VAT group was higher than that in the HepG2+VAT+EXE group, but there was no statistical difference (1.11 ± 0.08/GAPDH vs. 0.96 ± 0.12/GAPDH, p>0.05). Compared with the HepG2+SAT group, the expression of p-IRS2(S731) protein in the HepG2+SAT+EXE group was decreased, but the difference was also not statistically significant (0.97 ± 0.04/GAPDH vs. 0.75 ± 0.08/GAPDH, p>0.05) (**Figure 6A**).

The expression results of p-IRS2(S731) protein in HepG2 cells of each co-culture group after insulin stimulation will be revealed. Compared with HepG2+insulin group, the expression of p-IRS2(S731) protein in HepG2+VAT+insulin group, HepG2+SAT+insulin group and HepG2+VAT+EXE+insulin group were all increased. Although the expression of p-IRS2(S731) protein in HepG2+SAT+insulin group was lower than that in HepG2+VAT+insulin group (0.81 ± 0.07/GAPDH vs. 1.03 ± 0.06/GAPDH, p>0.05), there was no statistical difference. Compared with HepG2+VAT+insulin group, the expression of p-IRS2(S731) protein in HepG2+VAT+EXE+insulin group decreased by 23.87 ± 4.78% (1.03 ± 0.06/GAPDH vs. 0.78 ± 0.03/GAPDH, p<0.05). Compared with HepG2+SAT+insulin group, the expression of p-IRS2(S731) protein in HepG2+SAT+EXE+insulin group decreased by 52.16 ± 9.86% (0.81 ± 0.07/GAPDH vs. 0.39 ± 0.09/GAPDH, p<0.01) (**Figure 6B**).

#### 3.3.3 Exenatide promotes the expression of PI3K-p85 protein in HepG2 cells

**Figure 7:**
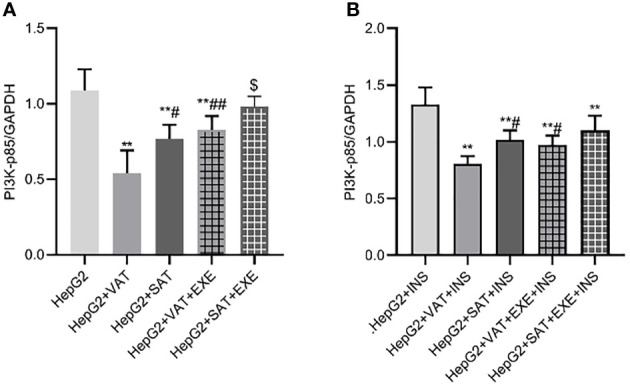
The influence of exenatide on the expression of PI3K-p85 protein in hepatocytes conducted by different regional adipose tissue. **(A)** Expression of PI3K-p85 protein in HepG2 cells of each group. Data are means ± SEM, n = 3/group, **P < 0.01 vs. HepG2 group; ^#^P < 0.05, ^##^P < 0.01 vs. HepG2+VAT group; ^$^P < 0.05 vs. HepG2+SAT group. **(B)** Expression of PI3K-p85protein in HepG2 cells of each group under the action of insulin. Data are means ± SEM, n = 3/group, **P < 0.01 vs. HepG2+INS group; ^#^P < 0.05 vs. HepG2+VAT+INS group.

Adipose tissue of different parts can inhibit the expression of PI3K-p85 protein in HepG2 cells. Specifically, compared with HepG2 group, the expression of PI3K-p85 protein in HepG2+VAT group (1.09 ± 0.14/GAPDH vs. 0.54 ± 0.15/GAPDH, p<0.01) and HepG2+SAT group (1.09 ± 0.14/GAPDH vs. 0.77 ± 0.09/GAPDH, p<0.01) was decreased. HepG2+VAT group inhibited PI3K-p85 protein expression more than HepG2+SAT group (0.54 ± 0.15/GAPDH vs. 0.77 ± 0.09/GAPDH, p<0.05). Furthermore, exenatide improved the inhibitory effect of adipose tissue on PI3K-p85 protein in HepG2 cells. Compared with the HepG2+VAT group, the expression of PI3K-p85 protein was increased in the HepG2+VAT+EXE group (0.54 ± 0.15/GAPDH vs. 0.82 ± 0.09/GAPDH, p<0.01). The protein of PI3K-p85 was also increased in the HepG2+SAT+EXE group compared with the HepG2+SAT group (0.99 ± 0.06/GAPDH vs. 0.77 ± 0.09/GAPDH, p<0.05) (**Figure 7A**).

To further explore the effect of insulin on PI3K-p85 protein, we added insulin to each co-culture group. Compared with HepG2+insulin group, the expression of PI3K-p85 protein in HepG2+VAT+insulin group, HepG2+SAT+insulin, HepG2+VAT+EXE+insulin group and HepG2+SAT+EXE+insulin group was decreased(p<0.01). Compared with HepG2+VAT+insulin group, the expression of PI3K-p85 in HepG2+SAT+insulin group (0.81 ± 0.06/GAPDH vs. 1.02 ± 0.08/GAPDH, p<0.05) and HepG2+VAT+EXE+insulin group (0.81 ± 0.06/GAPDH vs. 0.97 ± 0.08/GAPDH, p<0.05) were increased. Compared with HepG2+SAT+insulin group, the expression of PI3K-p85 protein in HepG2+SAT+EXE+insulin group was increased (1.02 ± 0.08/GAPDH vs. 1.10 ± 0.13/GAPDH, p>0.05), but there was no statistical difference (**Figure 7B**).

#### 3.3.4 Exenatide does not affect the expression of Akt2 protein in HepG2 cells

**Figure 8:**
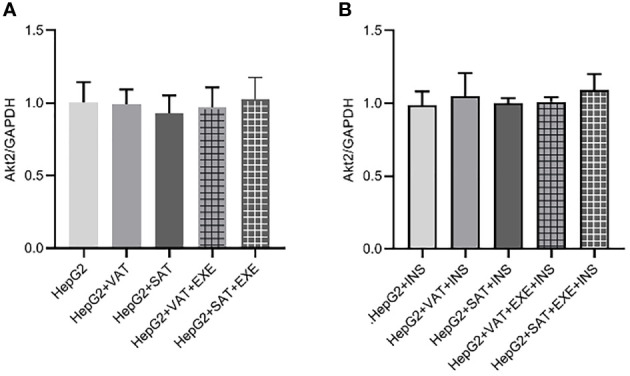
The influence of exenatide on the expression of Akt2 protein in hepatocytes conducted by different regional adipose tissue. **(A)** Expression of Akt2 protein in HepG2 cells of each group. Data are means ± SEM, n = 3/group. **(B)** Expression of Akt2 protein in HepG2 cells of each group under the action of insulin. Data are means ± SEM, n = 3/group.

There was no significant difference in Akt2 protein expression between HepG2 group, HepG2+VAT group, HepG2+SAT group, HepG2+VAT+EXE group, and HepG2+SAT+EXE group (**Figure 8A**).

We similarly added insulin to each co-culture group. The results also showed that there was no difference in the expression of AKt2 protein in HepG2+insulin, HepG2+VAT+insulin group, HepG2+SAT+insulin group, HepG2+VAT+EXE+insulin group and HepG2+SAT+EXE+insulin group (**Figure 8B**).

#### 3.3.5 Exenatide promotes the expression of p-Akt2(S473) protein in HepG2 cells

**Figure 9:**
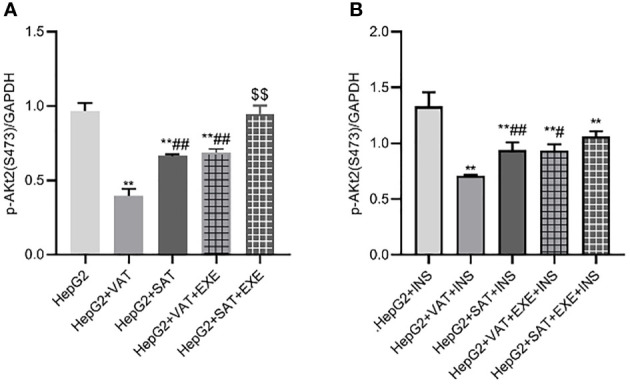
The influence of exenatide on the expression of p-AKt2(S473) protein in hepatocytes conducted by different regional adipose tissue. **(A)** Expression of p-AKt2(S473) protein in HepG2 cells of each group. Data are means ± SEM, n = 3/group, **P < 0.01 vs. HepG2 group; ^##^P < 0.01 vs. HepG2+VAT group; ^$$^P < 0.01 vs. HepG2+SAT group. **(B)** Expression of p-AKt2(S473) protein in HepG2 cells of each group under the action of insulin. Data are means ± SEM, n = 3/group, **P < 0.01 vs. HepG2+INS group; ^#^P < 0.05, ^##^P < 0.01 vs. HepG2+VAT+INS group.

Similar to the expression of IRS2 and PI3K-p85 protein, adipose tissue can also inhibit the expression of p-Akt2(S473) protein in HepG2 cells. Specifically, compared with HepG2 group, the expression of p-Akt2(S473) in HepG2+VAT group (0.96 ± 0.05/GAPDH vs. 0.40 ± 0.05/GAPDH, p<0.01) and HepG2+SAT group (0.96 ± 0.05/GAPDH vs. 0.66 ± 0.01/GAPDH, p<0.01) was decreased. HepG2+VAT group inhibited p-Akt2(S473) protein expression more than HepG2+SAT group (0.40 ± 0.05/GAPDH vs. 0.66 ± 0.01/GAPDH, p<0.01). Furthermore, exenatide improves the inhibitory effect of adipose tissue on p-Akt2(S473) protein in HepG2 cells. Compared with the HepG2+VAT group, the expression of p-Akt2(S473) protein was increased in the HepG2+VAT+EXE group (0.40 ± 0.05/GAPDH vs. 0.69 ± 0.03/GAPDH, p<0.01). The protein of p-Akt2(S473) was also increased in the HepG2+SAT+EXE group compared with the HepG2+SAT group (0.95 ± 0.06/GAPDH vs. 0.66 ± 0.01/GAPDH, p<0.05) (**Figure 9A**).

To further explore the effect of insulin on p-Akt2(S473) protein, we added insulin to each co-culture group. Compared with HepG2+insulin group, the expression of p-Akt2(S473) protein in HepG2+VAT+insulin group, HepG2+SAT+insulin, HepG2+VAT+EXE+insulin group and HepG2+SAT+EXE+insulin group was decreased (p<0.01). Compared with HepG2+VAT+insulin group, the expression of p-Akt2(S473) in HepG2+SAT+insulin group was higher (0.71 ± 0.01/GAPDH vs. 0.94 ± 0.07/GAPDH, p<0.01). The expression of p-Akt2(S473) protein in HepG2+VAT+insulin group was lower than that in HepG2+VAT+EXE+insulin group (0.71 ± 0.01/GAPDH vs. 0.93 ± 0.06/GAPDH, p<0.05). Compared with HepG2+SAT+insulin group, the expression of p-Akt2(S473) protein in HepG2+SAT+EXE+insulin group was no statistical difference (0.94 ± 0.07/GAPDH vs. 1.06 ± 0.05/GAPDH, p>0.05) (**Figure 9B**).

## 4 Discussion

This study investigated the effect of exenatide on key proteins of IRS2/PI3K/Akt2 signaling pathway in hepatocytes altered by different regional adipose tissue. We found that VAT secretes more IL-8, MCP-1, VEGF and sTNFR2 than SAT under the co-culture condition. Exenatide inhibited the secretion of these adipokines. In addition, adipose tissue can reduce the expression of IRS2, PI3K-p85, p-Akt2(S731) protein in HepG2 cells and increase the expression of IRS2(S731) protein in HepG2 cells. Exenatide restored insulin IRS2/PI3K/Akt2 signaling pathway impaired by adipose tissue. This study reveals a novel protective role for exenatide in hepatic IR induced by adipose tissue located at different regions. Exenatide may improve adipose tissue-associated hepatic IR by inhibiting adipokines and regulating the expression of key proteins in the IRS2/PI3K/Akt2 pathway.

### 4.1 Exenatide inhibits cytokines secreted by adipose tissue at different sites

Obesity is an increasing public health problem that leads to metabolic syndrome and increased risk of T2DM (25). The adipokines secreted by different parts of adipose tissue are different. By using adipokine antibody array (Raybiotech, ref. AAH-ADI-G1) our previous studies find that human VAT releases IL-8, MCP-1, VEGF and sTNFR2 more than SAT (unpublished data). The results of this study showed that VAT still released more IL-8, MCP-1, VEGF and sTNFR2 than SAT under co-culture conditions with HepG2 cells. IL-8, a novel leukocyte chemotactic activating cytokine, is produced by various types of cells upon stimulation with inflammatory stimuli and exerts a variety of functions on leukocytes, particularly, neutrophils *in vitro (* 26). Our findings are consistent with investigators Ritter A (27) and Koenen TB (28) that VAT releases more IL-8 than SAT.

MCP-1 is a chemokine secreted by macrophages, endothelial cells, preadipocytes, and mature adipocytes. MCP-1 attracts monocytes and T lymphocytes to the site of inflammation (29). One study found that serum MCP-1 levels elevated with increasing VAT in obese women, and MCP-1 was positively correlated with serum insulin level and degree of IR (30). The study of Kim WK et al. suggested that MCP-1 may be involved in the development of fat deposition and IR (31). This is consistent with our finding that VAT releases more MCP-1 than SAT *in vitro* under co-culture conditions with HepG2 cells.

The main function of VEGF is to induce the formation of new blood vessels. VEGF secreted by adipocytes can indirectly influence the inflammatory response by increasing vascular permeability (32). Lysaght J et al. found that VAT expresed a higher VEGF gene than SAT (33). Schlich R et al. also revealed that VAT secretes VEGF more than SAT, and VEGF can promote the proliferation of vascular smooth muscle cells (34). The above studies are consistent with our experimental results that VAT secretes more VEGF than SAT under co-culture conditions with HepG2 cells.

Serum levels of sTNFR1 and sTNFR2 in patients with acute alcoholic hepatitis are elevated, and serum sTNFR1 and sTNFR2 levels are closely related to liver inflammation and fibrosis (35). The concentration of sTNFR2 in the blood circulation will increase in obesity, and the effect of VAT on the concentration of sTNFR2 in the blood circulation is greater than that of SAT (36), which is also consistent with our experimental results.

GLP-1 is secreted by enteroendocrine L cells in response to oral nutrient intake and triggers glucose-stimulated insulin secretion while suppressing glucagon secretion. It also slows gastric emptying, which contributes to reduce postprandial glycemic excursions after meals (37). Exenatide is widely used in patients with T2DM who still have suboptimal glycemic control under oral metformin and sulfonylurea drugs (38). The study by Viswanathan P et al. demonstrated that exenatide was effective in treating obese patients with type 2 diabetes, resulting in weight loss and reductions in glycosylated hemoglobin (HbA_lc_), systolic blood pressure, triglycerides, and high-sensitivity C-reactive protein (CRP) (39). By using a dynamic positron emission tomography, researchers found exenatide improves both hepatic and adipose tissue IR (40). Our results showed that the concentrations of IL-8, MCP-1, VEGF and sTNFR2 in the HepG2+AT+EXE group were lower than those in the HepG2+AT group, suggesting that exenatide could reduce the secretion of adipokines in adipose tissue. This finding provides evidence to support the hypothesis that GLP-1RA may regulate the amounts of adipokines secreted by SAT and VAT.

### 4.2 Exenatide regulates the key proteins of IRS2/PI3K/Akt2 insulin signaling pathway in hepatocytes induced by different regional adipose tissue

#### 4.2.1 Exenatide improves the inhibitory effect of adipose tissue on IRS2 protein in hepatocytes

IRS2 is a key protein in the insulin signaling pathway. Epigenetic studies found that IRS2 was significantly downregulated in the liver of obese T2DM patients (41). Mice knocked out of the IRS2 gene develop T2DM, which is attributed to IRS2 deficiency leading to IR in the liver and reduced numbers of islet Β cell (42). Therefore, IRS2 plays a key role in glucose metabolism. We detected the expression of IRS2 protein in HepG2 cells and observed the effect of VAT and SAT on the expression of IRS2 protein with or without exenatide. The results showed that the IRS2 protein in the HepG2+AT group (HepG2+VAT group and HepG2+SAT group) were lower than that in the HepG2 group under both basal and insulin-stimulated conditions. Under basal culture conditions, the expression of IRS2 protein in the HepG2+VAT group was lower than that in the HepG2+SAT group. Under the condition of insulin stimulation, there was no significant difference in IRS2 protein expression between HepG2+VAT+insulin group and HepG2+SAT+insulin group, which may be related to insulin action. The above results suggested that both VAT and SAT can down-regulate the expression of IRS2 protein in HepG2 cells, and the effect of VAT is more significant than that of SAT.

In addition, the IRS2 protein in the HepG2+AT+EXE group was significantly higher than that in the HepG2+AT group. The IRS2 protein in the HepG2+SAT+EXE+insulin group was higher than that in the HepG2+SAT+insulin group under insulin-stimulated conditions. There was no significant difference in IRS2 protein expression between HepG2+VAT+EXE+insulin group and HepG2+VAT+insulin group. In conclusion, exenatide may improve adipose tissue-induced IR by promoting the expression of IRS2 protein in hepatocytes. The above results are consistent with the study by Kubota T et al., who pointed out that the expression of IRS2 protein in liver biopsy specimens of subjects with non-alcoholic fatty liver was significantly lower than that of subjects with non-fatty liver (43). Yang H et al. found that exenatide could improve IR in high-fat diet-fed mice and restore IRS1/2 levels that was down-regulated by high-fat diet (44).

#### 4.2.2 Exenatide improves the stimulatory effect of adipose tissue on p-IRS2(S731) protein in hepatocytes

IRS contains tyrosine and serine sites. The biological effects of phosphorylation of these two amino acids are different (45). Most of the current research focuses on the phosphorylation of IRS1. Insulin can activate the IRS kinase, which can phosphorylate the serine/threonine of IRS, thereby interrupting the binding of IRS to downstream effectors, resulting in IR (46, 47). Rector RS et al. found that high expression of p-IRS2 (S731) protein led to IR in mouse hepatocytes with mitochondrial dysfunction (48). We speculate that p-IRS2 (S731) protein may also be involved in the transduction of insulin signaling pathway. Our previous study found that both VAT and SAT could significantly up-regulate p-IRS2 (S731) protein expression, and VAT had a more significant effect on p-IRS2 (S731) protein expression than SAT. This is consistent with the results of our present study. On the other hand, the p-IRS2 (S731) protein in HepG2+AT+insulin group was higher than that in HepG2+insulin group. The p-IRS2 (S731) protein in HepG2+VAT+insulin group was slightly higher than that in HepG2+SAT+insulin group, but the difference was not significant. The results suggested that adipose tissue can increase the level of p-IRS2 (S731) protein, but the effects of VAT and SAT on p-IRS2 (S731) protein were not significant, which may be due to the small sample size.

Our experiment also showed that the p-IRS2 (S731) protein in the HepG2+AT+EXE group was lower than that in the HepG2+AT group in the basal state, but there was no statistical difference. After insulin stimulation, the p-IRS2 (S731) protein in the HepG2+AT+EXE+insulin group was lower than that in the HepG2+AT+insulin group, suggesting that exenatide may inhibit the expression of p-IRS2 (S731) protein elevated by adipose tissue. The expression levels of p-IRS2(S731) protein in the adipose tissue of high-fat diet-induced diabetic adipose tissue was significantly higher than that of the normal diet control group, but the expression levels of IRS2 protein in the liver and adipose tissue of the diabetic adipose tissue was lower than that of the control group (49). This is consistent with our findings. Therefore, it is reasonable to speculate that the function of hepatocyte IRS2 protein is different from that of p-IRS2(S731) protein. The IRS2 protein may be a participant in hepatic insulin signaling, and the p-IRS2(S731) protein may interfere with the normal insulin signaling transduction and lead to IR.

#### 4.2.3 Exenatide improves the inhibitory effect of adipose tissue on PI3K-p85 protein in hepatocytes

PI3K is one of the signaling molecules involved in intracellular signaling, which was first discovered by Whitman M (50). In addition, PI3Ks are divided into 3 subtypes based on substrate and structure specificity. One of the most widely studied is type I, which is directly activated by cell surface receptors. Type IA PI3K is a key mediator of hepatic insulin action, and it is a heterodimer composed of the regulatory subunit p85 and the catalytic subunit p110. When the extracellular insulin signal is transmitted into the cell through IRS, IRS binds to the SH2 domain of the regulatory subunit p85 of PI3K, thereby activating the catalytic subunit p110 of PI3K and catalyzing PIP2 to generate PIP3 (51). The results of this study showed that the PI3K-p85 protein in the HepG2+AT group was lower than that in the HepG2 group under both basal and insulin-stimulated conditions, and the PI3K-p85 protein in the HepG2+VAT group was lower than that in the HepG2+SAT group. It is suggested that the adipose tissue of different region can down-regulate the expression of PI3K-p85 protein, and the effect of VAT is more obvious than that of SAT. Similar results were documented in Ren C et al., who found that the expression of PI3K-p85 protein in rats with type 2 diabetes induced by high fat and streptozotocin was significantly lower than that of normal control rats.

The PI3K-p85 protein in the HepG2+AT+EXE group was higher than that in the HepG2+AT group in the basal state of this study. After insulin stimulation, the PI3K-p85 protein in the HepG2+VAT+EXE+insulin group was higher than that in the HepG2+VAT+insulin group. The PI3K-p85 protein of HepG2+SAT+EXE+insulin group was also higher than that of HepG2+SAT+insulin group, but there was no statistical difference. The sample size should be expanded in future studies to bring the results closer to the general state. The above results suggest that exenatide can improve PI3K-p85 protein expression reduced by adipose tissue. Similarly, Taniguchi CM et al. investigated the effect of PI3K depletion on hepatic glucose and lipid homeostasis. They found that mice with specific knockout of PI3K-p85 in the liver exhibited IR, hyperinsulinemia and hyperlipidemia (52). A synthetic peptide AWRK6 was found to attenuated diabetes as a novel GLP-1 receptor agonist candidate. In high energy diet-induced mice with non-alcoholic fatty liver disease, obesity and hepatic steatosis were alleviated by intraperitoneal injection of AWRK6. The phosphorylation of liver PI3K/AKT/AMPK/ACC was elevated significantly by AWRK6 administration (53). These studies, joined with our present findings, suggest that exenatide can increase the phosphorylation of PI3K in the liver and improve hepatic IR.

#### 4.2.4 Exenatide has no effect on the expression of Akt2 protein in HepG2 cells

Akt, as a major signaling protein downstream of PI3K, is a serine/threonine protein kinase, including three isoforms of Akt1, Akt2 and Akt3 (54). Akt1 is widely expressed in various tissues, while Akt2 is highly expressed in insulin-responsive tissues. Akt3 is only expressed in brain tissue and testis (55, 56). Multiple studies have demonstrated that mice knocked out of the Akt2 gene exhibit reduced Β-cell numbers and peripheral tissue IR (24, 57, 58). In addition, Akt2 gene mutations have also been observed in patients with severe IR and T2DM (59). However, in this part of our study, we found that there was no significant difference in Akt2 protein expression between the experimental groups. The results did not suggest that the effects of VAT and SAT on insulin sensitivity of hepatocytes were related to Akt2 protein and did not suggest that exenatide could affect the expression of Akt2 protein in hepatocytes. Gan KX et al. found that the expression of IRS2 protein in the liver of rats fed with a high-fat diet for 8 weeks decreased, but there was no significant change in the expression of Akt2 protein in the liver (60), which is consistent with our findings.

#### 4.2.5 Exenatide improves the inhibitory effect of adipose tissue on p-Akt2(S473) protein in hepatocytes

After PI3K-p85 binds to the corresponding receptor or receptor protein on the cell membrane, PI3K-p110 is activated and further catalyzes the generation of PIP3. PIP3 recruits PH domain containing PDK1 and AKt to the cell membrane. Subsequently, PDK1 phosphorylates the Thr308 site of the Akt catalytic domain, and PDK2 phosphorylates the Ser473 site of the Akt regulatory domain, resulting in full activation of AKt (61). Our previous study found that both VAT and SAT could significantly up-regulate p-IRS2 (S731) protein expression, and VAT had a more significant effect on p-IRS2 (S731) protein expression than SAT, which is consistent with the results of this study. Specifically, the p-AKt2(S473) protein in the HepG2+AT group was significantly lower than that in the HepG2 group, and the p-AKt2(S473) protein in the HepG2+VAT group was significantly lower than that in the HepG2+SAT group. After insulin stimulation, the p-AKt2(S473) protein in the HepG2+AT+insulin group was also lower than that in the HepG2+insulin group, and the p-AKt2(S473) protein in the HepG2+VAT+insulin group was lower than that in the HepG2+SAT+insulin group.

In addition, in the basal state, the p-AKt2(S473) protein in the HepG2+AT+EXE group was significantly higher than that in the HepG2+AT group. The results of this part suggest that exenatide can improve the expression of p-AKt2(S473) protein in HepG2 cells. Xing C et al. found that lipid deposition and Akt phosphorylation were reduced in the liver of high-fat diet-induced insulin-resistant rats. After 21 days of exenatide treatment, liver lipids and inflammatory cell infiltration were reduced, while Akt phosphorylation was increased (62). Some studies have found that exenatide can restore the phosphorylation of Akt in pancreatic Β cells downregulated by FFA and TNF-α (63, 64). The above studies are consistent with our experiments, suggesting that exenatide can improve IR by upregulating p-Akt, which is inhibited by adipose tissue.

## 5 Conclusions

In summary, the present study found that exenatide inhibited the inflammatory adipokines secretion of adipose tissue located in different regions and restored insulin IRS2/PI3K/Akt2 signaling transduction in hepatocyte. These results provided experimental evidence to explain the molecular mechanisms of GLP-1RA’s insulin-sensitising effects against adipose tissue-related IR in hepatocytes.

## Data availability statement

The original contributions presented in the study are included in the article/supplementary material. Further inquiries can be directed to the corresponding author.

## Ethics statement

The studies involving human participants were reviewed and approved by the Ethics and Research Committee of First affiliated hospital of Kunming Medical University. The patients/participants provided their written informed consent to participate in this study.

## Author contributions

CB- Conceptualization, methodology, writing-original draft. YW- Investigation, data curation, formal analysis, writing-original draft. YG- Investigation, data curation. JH-Investigation, data curation. XN- Investigation and resources, data curation. FZ- Investigation, data curation. JZ- Investigation, data curation. TK- Investigation, funding acquisition, writing-review and editing. BW- Supervision, writing-review and editing, funding acquisition BW supervised the study, had full access to all the data in the study and takes responsibility for the integrity of the data and the accuracy of the data analysis. All authors contributed to the article and approved the submitted version.

## Funding

This study was supported by Bethune-SanSheng Sunshine Diabetes Research Fund; The Yunnan Province Clinical Research Center for Metabolic disease (202102AA100056); KunmingMedical University Applied Basic Research Joint Project (202201AY0700001-072); The National Natural Science Fund of China (NSFC), under Award Number 81660141. The views in this paper are solely the authors’ responsibility and do not represent official views, either expressed or implied, of NSFC and the Chinese Government.

## Acknowledgments

We thank all participants for their dedicated and conscientious collaboration.

## Conflict of interest

The authors declare that the research was conducted in the absence of any commercial or financial relationships that could be construed as a potential conflict of interest.

## Publisher’s note

All claims expressed in this article are solely those of the authors and do not necessarily represent those of their affiliated organizations, or those of the publisher, the editors and the reviewers. Any product that may be evaluated in this article, or claim that may be made by its manufacturer, is not guaranteed or endorsed by the publisher.
